# Nosocomial Transmission of Dengue

**DOI:** 10.3201/eid1010.040464

**Published:** 2004-10

**Authors:** Zsuzsanna Nemes, Gabriella Kiss, Edit P. Madarassi, Zoltán Peterfi, Emoke Ferenczi, Tamas Bakonyi, Gabor Ternak

**Affiliations:** * County Hospital, Pécs, Hungary;; † Johan Bela National Center for Epidemiology, Budapest, Hungary;; ‡ University of Veterinary Medicine, Vienna, Austria;; § Szent Istvan University, Budapest, Hungary

**Keywords:** letter, flavivirus, nosocomial infection, virus identification

**To the Editor:** Four viruses form the dengue complex of mosquito-borne viruses (family *Flaviviridae*, genus *Flavivirus*). Any of these viruses can cause dengue fever, an uncomplicated febrile illness with rash; however, these viruses are not transmitted person to person. The principal mosquito vector of these viruses is *Aedes aegypti*. These viruses are not known to exist in Europe; therefore, dengue virus infections in Europe are seen in patients returning from dengue-endemic areas (*1*). Nosocomial transmissions of dengue viruses by needlestick have been reported in three instances (*2**–**4*) and by bone marrow transplant in one instance (*5*). We describe the first case of nosocomial dengue fever diagnosed and treated in Hungary.

On September 6, 2003, a 46-year-old physician sought care from the Department of Infectology, ("Baranya County Hospital" Pécs, Hungary); he reported a 4-day history of fever, headache, malaise, maculopapular rash, and pharyngitis. He had recently returned from a trip to Thailand and recalled having been bitten by a mosquito at Bangkok airport 11 days earlier. The patient had no history of illnesses before he left Hungary to go to Thailand. On examination, laboratory results indicated leukopenia (3,300 leukocytes/mm^3^) and mild thrombocytopenia (119,000 platelets/mm^3^). Leukopenia is characteristic of dengue virus and has been associated with suppression of bone marrow production (*6*). We conducted additional tests because thrombocytopenia could have been the first sign of a more severe form of dengue infection, dengue hemorrhagic fever, which is associated with hemorrhagic diathesis and shock (*6*). Lymphocytosis and monocytosis with 26% atypical lymphocytes and a high-normal level of alanine aminotransferase (56 U/L) were found. The C-reactive protein level and the erythrocyte sedimentation rate were normal. Blood smears for malarial parasites were negative.

Examination of the patient showed a maculopapular rash, pharyngitis, and conjunctivitis. Dengue fever was the clinical diagnosis based on the patient's history of a mosquito bite in a dengue-endemic country, the patient's symptoms, and the laboratory results. The patient's general condition was relatively good, so we treated him on an outpatient basis and recommended that he return for daily examinations.

On September 7, while collecting a blood sample from the patient, the patient's sister, also a physician, accidentally stuck her finger with the needle, which was contaminated with the patient's blood. Seven days later she became ill, with fever, headache, diffuse maculopapular rash, myalgia, cervical lymphadenopathy, and malaise. Her laboratory tests showed leukopenia with a normal thrombocyte level, C-reactive protein level, liver function tests, and erythrocyte sedimentation rate. On physical examination, painfully enlarged cervical lymph nodes and conjunctivitis were found. No complications were observed and the disease resolved within 10 days after onset in both patients. The female patient had never traveled to a dengue-endemic region.

Serologic and virologic evidence confirmed the clinical diagnosis. Acute-phase serum samples from each patient were tested for immunoglobulin (Ig) M and IgG antibodies to dengue viruses by using a commercial enzyme-linked immunosorbent assay kit. IgM, but not IgG, antibodies to dengue viruses were detected in the serum sample from the male patient 7 days after the onset of his illness; a convalescent-phase serum sample was not available for further testing. The first serum sample was obtained from the female patient 6 days after onset of her illness. IgM and IgG antibodies were not found in that sample. In the serum sample obtained from the female patient 12 days after onset, IgM, but not IgG, antibodies to dengue viruses were found. Both IgM and IgG antibodies were found in serum samples from this patient 3 weeks after onset of her illness.

Diagnosis was also confirmed by reverse transcription–polymerase chain reaction assays of early serum samples of both patients by using universal flavivirus primers. Amplification products were directly sequenced (GenBank accession no. AY538627 and AY538628). The nucleotide sequences were identified with a BLAST search (http://www.ncbi.nlm.nih.gov/BLAST/) using the GenBank database. Highest similarity was with dengue virus type 2 strain ThNH76/93, which had been isolated from a patient in northeast Thailand during the epidemic season of 1993 (*7*). The virus- specific nucleotide sequences detected in the Hungarian patients showed 98% nucleotide identity with the corresponding sequences of the Thai strain.

Viremia and simultaneous antibody production has been observed in several studies of dengue (*6**,**8**,**9*). Virus isolation is possible in dengue infections early in the illness, and in our experience, virus RNA was detected during the early febrile period. The male patient still had fever when the needle accident occurred, and the needle was contaminated.

Infectious disease specialists and other physicians should recognize that vector-borne diseases, such as dengue and malaria, are potentially life threatening. Therefore, they should consider these diseases in the differential diagnosis of febrile patients returning from tropical countries. In most patients, dengue fever resolves without hemoconcentration, an indication of dengue hemorrhagic fever. Nosocomial transmission of dengue viruses is not a common event, however, physicians must consider these diseases.

These unique cases demonstrate the possible introduction and transmission of exotic tropical viruses in a country within temperate zones; all that is needed are competent vectors. Whereas *A. aegypti* is not endemic in Europe, it could be introduced. The *A. albopictus* mosquitoes, an invader from Asia, already exists there, albeit in isolated areas (*10*). Patients returning from distant regions should be treated with increased attention and care. Although dengue viruses are rarely transmitted person to person, this incident emphasizes the importance of having reliable and rapid diagnostic methods available for early detection of imported infections with exotic viral agents.
